# Enhancing security in instant messaging systems with a hybrid SM2, SM3, and SM4 encryption framework

**DOI:** 10.1371/journal.pone.0332665

**Published:** 2025-09-15

**Authors:** He-Jun Lu, Roben A. Juanatas, Mideth B. Abisado

**Affiliations:** 1 College of Computing and Information Technologies, National University, Manila, Philippines; 2 The School of Big Data and Artificial Intelligence, Anhui Xinhua University, Hefei, Anhui, China; Jaramogi Oginga Odinga University of Science and Technology, KENYA

## Abstract

With the rapid integration of instant messaging systems (IMS) into critical domains such as finance, public services, and enterprise operations, ensuring the confidentiality, integrity, and availability of communication data has become a pressing concern. Existing IMS security solutions commonly employ traditional public-key cryptography, centralized authentication servers, or single-layer encryption, each of which is susceptible to single-point failures and provides only limited resistance against sophisticated attacks. This study addresses the research gap regarding the complementary advantages of SM2, SM3, and SM4 algorithms, as well as hybrid collaborative security schemes in IMS security. This paper presents a hybrid encryption security framework that combines the SM2, SM3, and SM4 algorithms to address emerging threats in IMS. The proposed framework adopts a decentralized architecture with certificateless authentication and performs all encryption and decryption operations on the client side, eliminating reliance on centralized servers and mitigating single-point failure risks. It further enforces an encrypt-before-store policy to enhance data security at the storage layer. The framework integrates SM2 for key exchange and authentication, SM4 for message encryption, and SM3 for integrity verification, forming a multi-layer defense mechanism capable of countering Man-in-the-Middle (MITM) attacks, credential theft, database intrusions, and other vulnerabilities. Experimental evaluations demonstrate the system’s strong security performance and communication efficiency: SM2 achieves up to 642 times faster key generation and 2.2 times faster decryption compared to RSA-3072; SM3 improves hashing performance by up to 11.5% over SHA-256; and SM4 delivers up to 22% higher encryption efficiency than AES-256 for small data blocks. These results verify the proposed framework’s practicality and performance advantages in lightweight, real-time IMS applications.

## Introduction

During the digital transformation of Chinese society, IMS have progressively evolved from their initial social functions to encompass public service domains such as financial transactions, government services, and enterprise organizational management. Furthermore, they have catalyzed the emergence of new market models like community group buying. As a vital component of digital infrastructure for social governance, an essential driving force behind economic transformation, and a key facilitator of public welfare services, IMS has demonstrated irreplaceable value in contemporary society.

With the rapid surge in China’s internet user base and the widespread adoption of IMS, information security concerns have become increasingly severe. According to the Statistical Report on Internet Development in China, by December 2024, the number of internet users in China had reached 1.108 billion, with IMS users accounting for 1.081 billion, representing 97.6% of the total internet population [1]. During IMS usage, users frequently encounter security threats, including stolen account credentials [2,3], leaked personal information [4], and intercepted or compromised chat records [5,6], highlighting the inadequacy of current security mechanisms in mitigating emerging cyber threats [7]. With the increasing complexity of IMS functionalities and the diversification of their applications, traditional encryption techniques struggle to meet emerging security demands, particularly in defending against MITM attacks, data tampering, and unauthorized access. Most IMS security solutions available on the market today primarily rely on centralized encryption mechanisms, which can make servers prime targets for cyberattacks and pose a risk of single points of failure [8,9]. In contrast, decentralized security solutions reduce dependence on a central server, thereby mitigating potential security risks. Therefore, the development of an efficient and secure encryption framework to enhance IMS data protection has emerged as a critical and urgent challenge.

At present, there is an important gap in the research of security schemes for IMS: No complete solution that can give full play to the collaborative advantages of SM2, SM3, and SM4 algorithms has emerged yet. The current literature lacks an in-depth exploration of end-to-end IMS security architectures. Consequently, this article proposes an efficient and secure hybrid encryption security framework for IMS, which integrates decentralization, certificateless authentication, and end-to-end encrypted transmission based on the complementary strengths of the SM2, SM3, and SM4 algorithms. This framework will be capable of effectively mitigating multiple security threats, such as data breaches, MITM attacks, and database intrusions, thereby significantly enhancing the security and reliability of IMS.

The main contributions of this study are summarized as follows:

- **Decentralized security framework with certificateless authentication:** We propose a novel security framework based on a decentralized architecture, which fully delegates encryption and decryption operations to client devices. This approach eliminates reliance on a central server and reduces the risk of single-point failures inherent in traditional architectures.
- **End-to-end hybrid encryption:** The framework implements a hybrid encryption mechanism throughout the transmission chain, ensuring data confidentiality, integrity, and availability during communication.
- **Encrypt-before-store principle:** All data are encrypted prior to storage in the database, thereby enhancing security at rest.
- **Hybrid encryption scheme for IMS:** To meet the real-time and low-latency requirements of Instant Messaging Systems (IMS), we implement a hybrid encryption and decryption scheme combining the SM2, SM3, and SM4 algorithms.
- **Performance evaluation against standard algorithms:** We evaluate the performance of SM2, SM3, and SM4 against conventional algorithms (RSA-3072, SHA-256, and AES-256) for small data blocks (less than or equal to 128 KB). Experiments were conducted using the Bouncy Castle cryptography library in a standardized environment with hardware acceleration for AES-NI and SHA-NI disabled.

The remainder of this paper is organized as follows: The Related work section systematically reviews the research progress on the application of SM2, SM3, and SM4 algorithms in the field of information security, and at the same time conducts a literature review on the relevant research achievements of IMS network security protection technology. The Methods section presents the proposed methodology based on SM2, SM3, and SM4 algorithms, introducing the overall security framework and the comprehensive security architecture alongside a hybrid encryption scheme for data communication and its implementation approach. The Results section details the experimental results obtained from this study. The Discussion section provides an analysis and discussion of the experimental findings. The Conclusions section concludes this paper by summarizing the key findings and contributions presented herein.

### Related work

China has made significant progress in cryptographic research, with SM2 [10], SM3 [11], and SM4 [12] recognized as international standards. Li et al. [13] proposed a side-channel-resistant SM2 point multiplication, while Zhai et al. [14] developed a distributed SM2 decryption scheme for IoT. Cao et al. [15] applied SM2 to trusted metrological data, and Zhang et al. [16] introduced traceable ring signatures. Wu et al. [17], Xu et al. [18], and Zhao et al. [19] optimized SM2-based authentication and key exchange. Jayakumari et al. [20] employed ECC for multimedia protection, and Prabhu et al. [21] enhanced cloud storage security. Compared with RSA, SM2 achieves superior efficiency, with a 256-bit key providing security equivalent to a 3072-bit RSA key [22,23]. However, these studies primarily focus on isolated improvements rather than integrated frameworks ensuring confidentiality, integrity, and availability.

Research on hash algorithms highlights SM3’s role as a robust alternative. Zheng et al. [24] designed a low-power SM3 implementation for IoT, and Han et al. [25] proposed a CUDA-based optimization to improve throughput. Stevens et al. [26] demonstrated chosen-prefix collisions for MD5, while Leurent and Peyrin [27] reported the first chosen-prefix collision on SHA-1, rendering legacy schemes unsuitable for secure applications. Nevertheless, prior work remains limited to algorithmic optimization or cryptanalysis, without system-level integration into low-latency secure communication frameworks.

Studies on SM4 have primarily focused on efficiency and adaptability. Guo et al. [28] extended SM4 into tweakable block ciphers, Zhang et al. [29] achieved record-breaking bit-sliced performance on x86, and Hu et al. [30] analyzed the SM algorithm family and software performance trade-offs. These contributions confirm SM4’s potential but do not explore system-level integration for latency-sensitive applications.

In instant messaging (IM) security, Liu et al. [31] implemented a hybrid 3DES–RC4 scheme. Tajudeen et al. [32] reviewed AES-based techniques for message protection. Kasar et al. [33] investigated decentralized WebRTC-based messaging, and Zhou et al. [34] designed an enterprise IM system with hierarchical protection. While these studies improve security and performance, they are largely confined to single algorithms or enterprise-specific solutions, leaving a gap in decentralized, end-to-end secure frameworks.

To address this gap, this study proposes a decentralized hybrid encryption framework integrating SM2, SM3, and SM4 with certificateless authentication. The framework enforces encrypt-before-store, mitigates single-point failures, and provides confidentiality, integrity, and availability for real-time IM systems, bridging the gap between algorithmic enhancements and practical deployment.

## Methods

### SM2 algorithm principle

**Fig 1:**
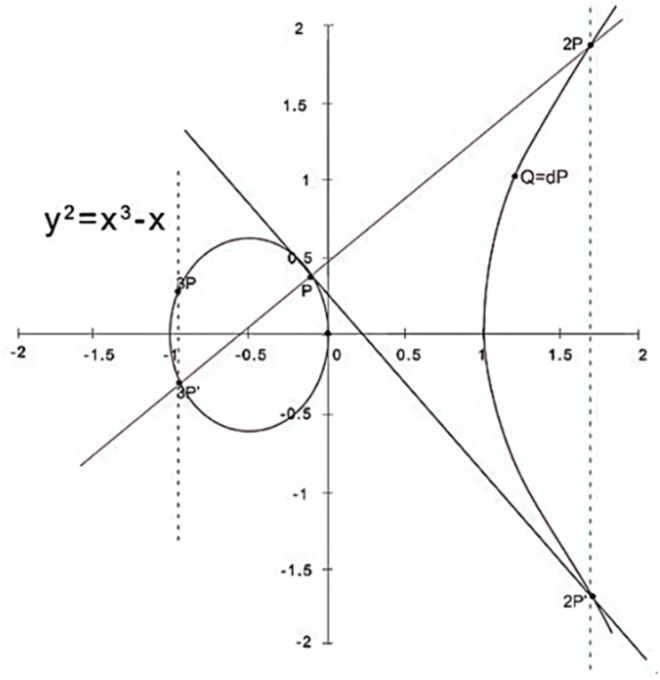
The principles of the SM2 algorithm.

SM2 is a public key cryptosystem that relies on the computational hardness of the Elliptic Curve Discrete Logarithm Problem (ECDLP). Let Q and P be two points on an elliptic curve, where Q=dP(d∈Z), d represents the private key, while Q serves as the corresponding public key. The challenge of deriving d from Q and P is considered computationally infeasible, forming the foundation of the algorithm’s security. The operational principles of the SM2 algorithm are depicted in Fig 1.

The encryption and decryption processes of the SM2 algorithm are defined as follows:

Consider a scenario where User A transmits a message M to User B, with L denoting the bit length of M. Let d be the private key, where d∈[1,n−1], and d is a randomly generated 256-bit integer. The parameter n represents the order of the publicly known base point G on the elliptic curve, while P denotes the corresponding public key, as formulated in Eq (1).


P=d×G (1)

Step A1: User A receives the public key P_B_ from User B, where P_B_ denotes a point on the elliptic curve, as defined in Eq (2).


$$P_{B}=\left(x_{B}{,y}_{B}\right)$$ (2)

Step A2: User A selects a random integer *k*, where k∈[1,n−1], and computes C_1_, as defined in Eq (3).


$${\text{C}}_{\text{1}}=\text{k}\times \text{G}=({\text{x}}_{\text{1}},{\text{y}}_{\text{1}})$$ (3)

Step A3: Compute the shared secret key S, as defined in Eq (4).


$$\text{S}=\text{k}\times {\text{P}}_{\text{B}}=({\text{x}}_{\text{s}},{\text{y}}_{\text{s}})$$ (4)

Step A4: Utilize the Key Derivation Function (KDF) to compute the encryption key t, as defined in Eq (5).


$$\text{t}=\text{KDF}({\text{x}}_{\text{s}}\left|\right|{\text{y}}_{\text{s}},\text{L})$$ (5)

Step A5: Compute the ciphertext C_2_, as defined in Eq (6), where ⊕ denotes the XOR operation.


$${\text{C}}_{\text{2}}=\text{M}\;⊕\;\text{t}$$ (6)

Step A6: Compute the ciphertext C_3_, as defined in Eq (7).


$${\text{C}}_{\text{3}}=\text{Hash}({\text{x}}_{\text{s}}\left||\text{M}|\right|{\text{y}}_{\text{s}})$$ (7)

Step A7: Compute the ciphertext C, as expressed in Eq (8).


$$\text{C}=({\text{C}}_{\text{1}}\left|\right|{\text{C}}_{\text{2}}\left|\right|{\text{C}}_{\text{3}})$$ (8)

Upon receiving the ciphertext C, User B extracts C_1_, C_2_, and C_3_.

Step B1: User B computes the shared secret key S′ using their private key d_B_, as defined in Eq (9).


$$\begin{array}{c} {\text{S}}^{\prime }={\text{d}}_{\text{B}}\times {\text{C}}_{\text{1}}=({\text{x}}_{\text{s}}\prime ,{\text{y}}_{\text{s}}\prime ) \end{array}$$ (9)

Step B2: Apply the KDF to compute the key t′, as defined in Eq (10).


$$\begin{array}{c} {\text{t}}^{\prime }=\text{KDF}({\text{x}}_{\text{s}}\prime ||{\text{y}}_{\text{s}}\prime ,\text{L}) \end{array}$$ (10)

Step B3: Decrypt C_2_ to obtain M′, as defined in Eq (11).


$$\begin{array}{c} {\text{M}}^{\prime }={\text{C}}_{\text{2}}\;⊕\;{\text{t}}^{\prime } \end{array}$$ (11)

Step B4: Compute C_3_′ as defined in Eq (12). If C_3_ is equal to C_3_′, then the plaintext M′ is successfully output.


$$\begin{array}{c} {\text{C}}_{\text{3}}\prime =\text{Hash}({\text{x}}_{\text{s}}^{\prime }\left|\right|{\text{M}}^{\prime }\left|\right|{\text{y}}_{\text{s}}\prime ) \end{array}$$ (12)

### SM3 cryptographic hash algorithm

**Fig 2:**
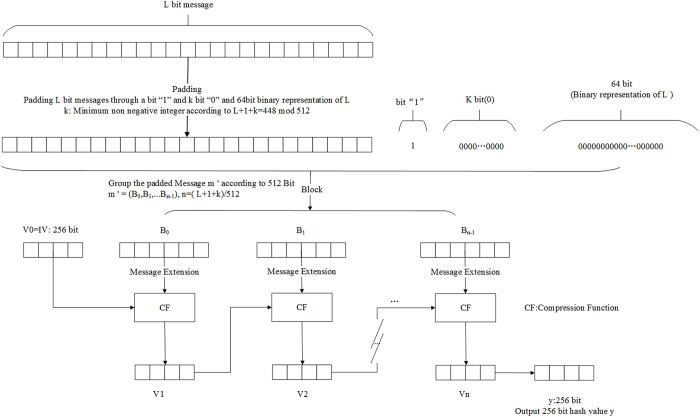
The SM3 cryptographic hash algorithm.

For a message m of length L (where $\text{L}<2^{64}$ bits), the SM3 hash algorithm processes the message in blocks and iteratively compresses it to generate a hash value of 256 bits. The algorithm flow is shown in Fig 2.

The message padding process begins by appending a ‘1’ bit, followed by k ‘0’ bits where k is the integer satisfying Eq (13) and a 64-bit L’s binary representation, thereby generating a padded message m’ with a length multiple of 512 bits as specified in Eq (14). The message is divided into n blocks (n determined by Eq 15), each denoted as B_i_. During computation, each block undergoes message expansion and is processed through the compression function CF in an iterative manner. As shown in Eq (16), V_n_ is the result of iterative compression, where V_0_ is the 256-bit initial value IV, ultimately producing a 256-bit hash value.


L+1+k≡448 mod 512 (13)

$$\begin{array}{c} {\text{m}}^{\prime }=({\text{B}}_{\text{0}},{\text{B}}_{\text{1}},\cdot \cdot \cdot ,{\text{B}}_{\text{n}-\text{1}}) \end{array}$$ (14)

n=(L+k+65)/512 (15)

$${\text{V}}_{\left(\text{i}+\text{1}\right)}=\text{CF}({\text{V}}_{\text{i}},{\text{B}}_{\text{i}})(\text{i}=\text{0},\text{1},\text{2}\cdot \cdot \cdot ,\text{n}-\text{1})$$ (16)

### SM4 block cipher algorithm

**Fig 3:**
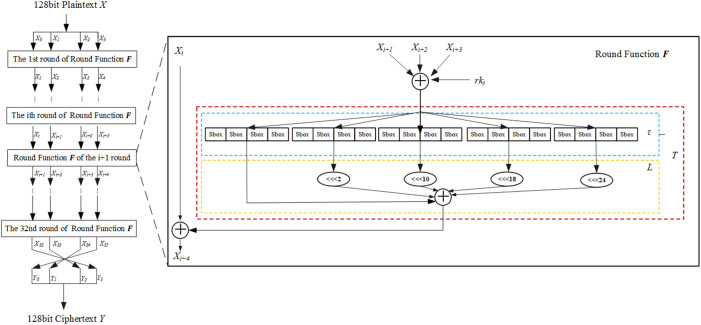
SM4 cryptographic algorithm flow chart.

SM4 is a block cipher characterized by a block size and key length of 128 bits. It utilizes an unbalanced Feistel structure and performs 32 iterations of the round functions during both the encryption and key expansion processes. The decryption process is designed to mirror that of encryption, with the round keys applied in reverse order. SM4 algorithm flow chart as shown in Fig 3.

Symbols and acronyms indicate meanings:

⊕: Denotes the bitwise exclusive OR operation performed on 32-bit words;

<<< i: Represents a circular left rotation by i bits.

Let X_0_, X_1_, X_2_, X_3_ be the round inputs and rk the round key. Then, F is defined as in Eq. (17).


$$\text{F}\left({\text{X}}_{\text{0}},{\text{X}}_{\text{1}},{\text{X}}_{\text{2}}{,\text{X}}_{\text{3}},\text{rk}\right)={\text{X}}_{\text{0}}\;⊕\;\text{T}\left({\text{X}}_{\text{1}}\;⊕\;{\text{X}}_{\text{2}}\;⊕\;{\text{X}}_{\text{3}}\;⊕\;\text{rk}\right)$$ (17)

T is an invertible transformation consisting of a nonlinear part τ and a linear part L. τ uses four parallel S-boxes. For input A = (a_0_, a_1_, a_2_, a_3_), the output B = (b_0_, b_1_, b_2_, b_3_) is shown in Eq (18).


$$\text{B}=\tau \left(\text{A}\right)=(\text{Sbox}({\text{a}}_{\text{0}}),\text{Sbox}({\text{a}}_{\text{1}}),\text{Sbox}({\text{a}}_{\text{2}}),\text{Sbox}({\text{a}}_{\text{3}}))$$ (18)

The output from the nonlinear transformation τ is the input to the linear transformation L. Suppose the input to L is B, and the corresponding output is C, as shown in Eq (19).

The output of τ serves as the input to the linear transformation L. Let B be the input to L, and C the corresponding output, as defined in Eq (19).


C=L(B)=B⊕(B<<<2)⊕(B<<<10)⊕(B<<<18)⊕(B<<<24) (19)

The encryption process performs 32 iterations of the round function F, followed by a final reverse transformation R. The corresponding output ciphertext is (Y_0_, Y_1_, Y_2_, Y_3_) as shown in Eq (20).


$$\left({\text{Y}}_{\text{0}},{\text{Y}}_{\text{1}},{\text{Y}}_{\text{2}},{\text{Y}}_{\text{3}}\right)=\text{R}\left({\text{X}}_{\text{32}},{\text{X}}_{\text{33}},{\text{X}}_{\text{34}},{\text{X}}_{\text{35}}\right)=\left({\text{X}}_{\text{35}},{\text{X}}_{\text{34}},{\text{X}}_{\text{33}},{\text{X}}_{\text{32}}\right)$$ (20)

### Overall security architecture

**Fig 4:**
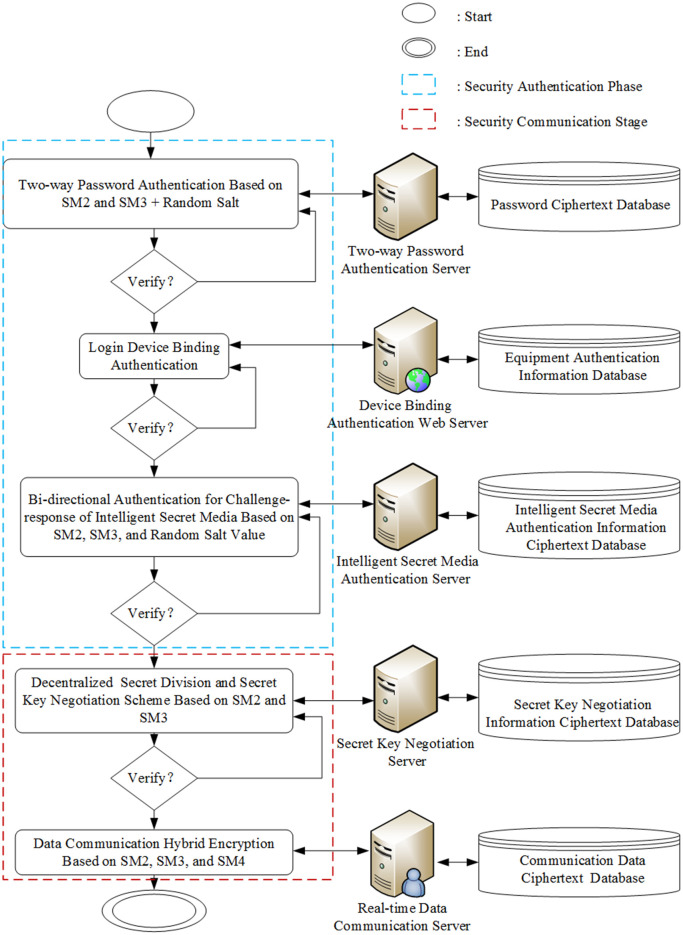
The overall security architecture.

Based on the security requirements of IMS, this paper proposes a highly confidential and secure communication framework based on SM2, SM3, and SM4 algorithms, including system security authentication, data security communication, and data security storage. This paper presents a hybrid encrypted communication framework based on SM series algorithms, which facilitates the secure transfer, storage, and validation of ciphertext throughout the communication process. The framework is designed to defend against various security threats, including unauthorized access, replay attacks, eavesdropping, cryptographic cracking, and MITM attacks. The overall system framework is depicted in Fig 4.

### A decentralized communication framework with hybrid encryption based on SM-series cryptographic algorithms

**Fig 5:**
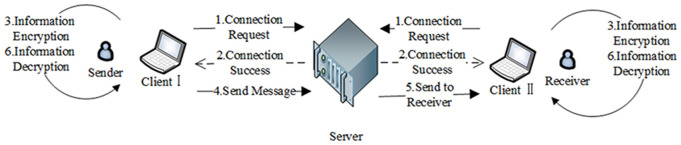
The schematic diagram of Encrypted Socket Communication.

To guarantee the security of communication between the client and the server, this paper designs a decentralized socket-encrypted communication framework based on the SM hybrid algorithm. Encrypted Socket technology is adopted for network communication between the server and the client. The communication process is realized through sockets. The socket operating on the client side is referred to as the Client Socket, while the one on the server side is called the Server Socket. Fig 5 illustrates the communication principle of the Socket encryption framework.

**Fig 6:**
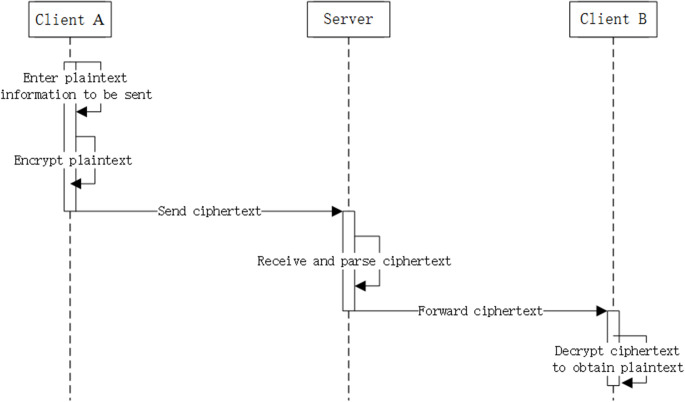
End-to-end communication timing diagram.

After client A successfully establishes a connection with client B, it subsequently acquires client B’s public key and prepares the data for transmission. Client A encrypts the data and transmits it to the server. The server then forwards the encrypted information to client B, who decrypts it layer by layer until obtaining the plaintext information. The same process applies when client B sends a message to client A. The sequence diagram for forwarding and transmitting information data between client A, client B, and the server is shown in Fig 6.

### A hybrid encryption scheme for data communication based on SM2, SM3, and SM4 algorithms

**Fig 7:**
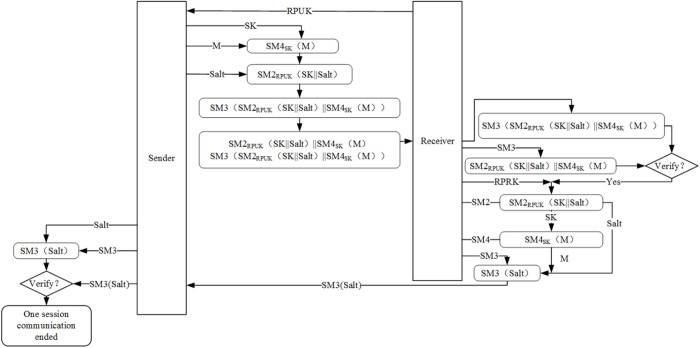
The proposed hybrid encryption scheme for data communication is based on SM2, SM3, and SM4.

After successful dual authentication between the client and server, the client is granted authorization to access the server. To ensure secure data communication among users, this paper proposes a decentralized end-to-end encrypted communication framework based on a hybrid SM algorithm. In this framework, data encryption and decryption are performed exclusively on the client side, while the server-side solely handles information forwarding, storage, and management tasks. Additionally, it facilitates ciphertext transmission and integrity verification throughout the entire process. The framework employs a symmetric SM4 algorithm along with SK for encrypting communication data; asymmetric algorithm SM2 is utilized for encrypting SK as well as random Salt authentication; furthermore, the SM3 algorithm is employed for verifying data integrity and random Salt authentication during data communication. The secure data communication framework based on a hybrid SM algorithm is shown in Fig 7.

**Fig 8:**
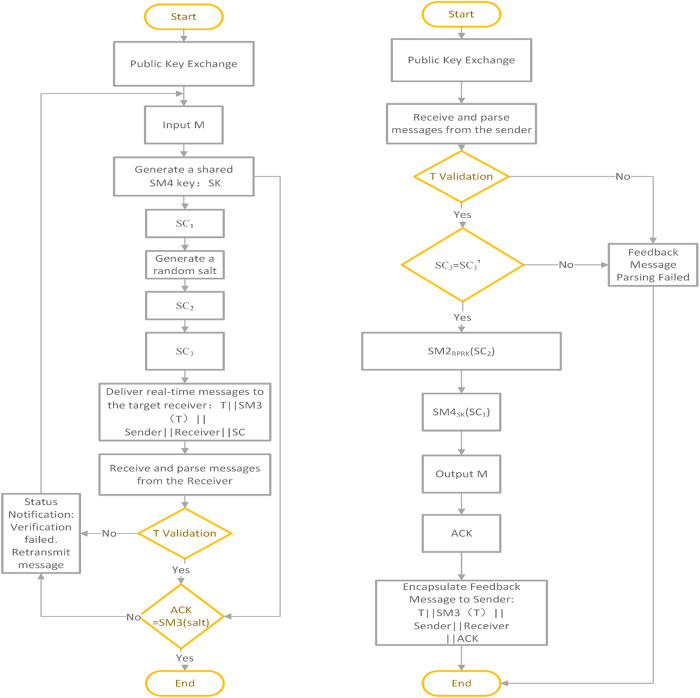
End-to-end data transmission protocol flowchart.

In the context of data communication, this study integrates the SM2, SM3, and SM4 algorithms to design a hybrid encryption-based secure communication framework utilizing the SM cryptographic algorithm suite. The SM2 asymmetric cryptographic algorithm is employed to ensure the security of the key, specifically encrypting the SK of SM4. The SM4 block cipher algorithm is primarily used for encrypting communication data, while the SM3 hash algorithm is responsible for ensuring data integrity verification. The notations and descriptions used in this framework are summarized in Table 1. Prior to communication between clients, the sender must first obtain the recipient’s public key. Using the SM2 algorithm, the sender encrypts both the SK and a randomly generated salt value with the recipient’s public key. These encrypted components, along with verification information and the ciphertext block, are transmitted to the recipient, thereby achieving key exchange, encrypted data transmission, and data integrity verification. Upon receiving the encrypted key block and ciphertext block, the recipient decrypts the key block using their SM2 private key to retrieve the SK. This SK is then used in conjunction with the SM4 algorithm to decrypt the ciphertext block, restoring the original plaintext message. Finally, the recipient computes SM3(Salt) and sends it back to the sender for session verification, ensuring the integrity and authenticity of the communication. The SM2 public key of the receiver is denoted as RPUK, and the receiver’s private key is RPRK. The plaintext information is denoted as M. Initially, the sender obtains the receiver’s public key. The flowchart of the end-to-end data transmission protocol is presented in Fig 8.

**Table 1:** The identifiers and descriptions used in the framework.

Identifier	Interpretative Statement
**Sender**	The client that sends messages
**Receiver**	The client that receives messages
**SK**	Session key of SM4
**M**	Messages
**RPUK**	Receiver public key
**RPRK**	Receiver private key
**Salt**	Random salt value
**T**	Timestamp

**Sender main steps:**

Step S1: Sender generates the SK.

Step S2: The Sender generates the ciphertext block SC_1,_ as shown in Eq (21).


$${\text{SC}}_{\text{1}}={\text{SM4}}_{\text{sk}}(\text{M})$$ (21)

Step S3: Sender generates a random Salt value.

Step S4: The sender embeds the SK into the random Salt value and encrypts it using the SM2 algorithm and the receiver’s public key (RPUK) to generate the ciphertext block SC_2_, as shown in Eq (22).


$${\text{SC}}_{\text{2}}={\text{SM2}}_{\text{RPUK}}\left(\text{SK}||\text{Salt}\right)$$ (22)

Step S5: The sender uses the SM3 algorithm to generate the ciphertext block SC_3_ as shown in Eq (23).


$${\text{SC}}_{\text{3}}={\text{SM3}({\text{SM4}}_{\text{sk}}\left(\text{M}\right)||\text{SM2}}_{\text{RPUK}}\left(\text{SK}||\text{Salt})\right)$$ (23)

Step S6: The sender sends the ciphertext block SC, as shown in Eq (24), to the receiver.


$$\text{SC}={\text{SC}}_{\text{1}}\left|\right|{\text{SC}}_{\text{2}}\left|\right|{\text{SC}}_{\text{3}}$$ (24)

**Receiver main steps:**

Step R1: The receiver obtains the ciphertext block SC and extracts SC_1,_ SC_2_, and SC_3_ separately. The receiver verifies it using the SM3 algorithm, calculating SC_3_’ as shown in the Eq (25). If SC_3_ = SC_3_’, the verification is successful.


$${\text{SC}}_{\text{3}}^{\prime }=\text{SM3}({\text{SC}}_{\text{1}}\left|\right|{\text{SC}}_{\text{2}})$$ (25)

Step R2: The receiver uses the RPRK of the SM2 algorithm to decrypt the SC_2_ block, obtaining the value SK || Salt, as shown in Eq (26), and then extracts the Salt and SK values using the Salt separation algorithm.


$$\text{SK}||\text{Salt}={\text{SM2}}_{\text{RPRK}}({\text{SC}}_{\text{2}})$$ (26)

Step R3: The receiver uses the SM4 algorithm and SK to decrypt the ciphertext block SC_1_ and obtain the plaintext M, as shown in Eq (27).


$$\text{M}={\text{SM4}}_{\text{sk}}({\text{SC}}_{\text{1}})$$ (27)

Step R4: The receiver sends an acknowledgment (ACK) confirmation message to the sender, as shown in Eq (28).


ACK=SM3(Salt) (28)

Finally, the sender uses the SM3 algorithm and Salt to compute SM3(Salt), which is then compared with the received ACK for verification. If the verification is successful, the session process concludes; otherwise, the session fails.

### Experimental environment

All experiments were conducted on a Windows 11 Professional workstation with an Intel Core i9-9880H CPU and 32 GB DDR4 RAM. The encryption algorithms were implemented in Java (JDK 17) with IntelliJ IDEA 2024.1.7 (Ultimate Edition) and the Bouncy Castle cryptography library. Hardware acceleration instructions (AES-NI and SHA-NI) were disabled to ensure consistent measurement results.

## Results

### The results of confidentiality, integrity, and availability testing

In the experiment, the memory values of the receiver and sender are obtained, and the packets of the sender and receiver are captured and analyzed by Wireshark, a packet capture and analysis tool, to obtain the communication data. The experimental results of the hybrid encryption scheme for data communication based on SM2, SM3, and SM4 in the proposed framework are presented in Table 2.

**Table 2:** The test results of the hybrid encryption scheme for data communication.

Data block name	Length (byte)	Data (hex)
**Sender’s plaintext data: M**	60	48656c6c6f2c2074686973206973206d792062616e6b206163636f756e742e20506c65617365206b65657020697420636f6e666964656e7469616c21
**SK**	16	D65F878CE8E6DC3F2428382540845DDE
**SC**_**1**_	64	4361E9EE86FA4828E80EB2C39A418351417DA3E558A447C98E7C9A9E31D7A2D8CEEA034ED01C6EB35D8E9F9158B466808A7C902E6F7DB95DC2D6E332CB5F2BC4
**Salt**	16	e9e679e5b90a03fd918248f51f27d265
**RPUK**	64	d490b5099d99b2ae4b4d7941ea341b59bd960726b21a9296176947063dcc4edccf1001590e64ff30d038544258688aa36d70c3ccd546534f9659b75a20d96eff
**SC**_**2**_	152	d2f3ce73717fc67c0ebd0938ddca2d76611b7726fcae1 cd6fcf8d3d97c5d7ed206eacbf5adbbe188dc319f9711d511d418fd38f4f8679d0358c854578c26ebf49daee3fb6129564dbbfe2af934e7cd6abe198dbfab097a168fbf36131bec99aba13c04f30c5ffae0a26bc22de38c5de03b9b13ce5369d5e1917d98d55f76c9fb7e02cfde79b5a34f7ff4888f7c37b4629028ad3632faccef
**SC**_**3**_	32	79202867054278d7651ea824b16e4440156846c1b6c17e34248f4907f4f077a5
**Sender’s ciphertext data: SC**	248	4361E9EE86FA4828E80EB2C39A418351417DA3E558A447C98E7C9A9E31D7A2D8CEEA034ED01C6EB35D8E9F9158B466808A7C902E6F7DB95DC2D6E332CB5F2BC4d2f3ce73717fc67c0ebd0938ddca2d76611b7726fcae1 cd6fcf8d3d97c5d7ed206eacbf5adbbe188dc319f9711d511d418fd38f4f8679d0358c854578c26ebf49daee3fb6129564dbbfe2af934e7cd6abe198dbfab097a168fbf36131bec99aba13c04f30c5ffae0a26bc22de38c5de03b9b13ce5369d5e1917d98d55f76c9fb7e02cfde79b5a34f7ff4888f7c37b4629028ad3632faccef79202867054278d7651ea824b16e4440156846c1b6c17e34248f4907f4f077a5
**Ciphertext data in transmission**	248	4361E9EE86FA4828E80EB2C39A418351417DA3E558A447C98E7C9A9E31D7A2D8CEEA034ED01C6EB35D8E9F9158B466808A7C902E6F7DB95DC2D6E332CB5F2BC4d2f3ce73717fc67c0ebd0938ddca2d76611b7726fcae1 cd6fcf8d3d97c5d7ed206eacbf5adbbe188dc319f9711d511d418fd38f4f8679d0358c854578c26ebf49daee3fb6129564dbbfe2af934e7cd6abe198dbfab097a168fbf36131bec99aba13c04f30c5ffae0a26bc22de38c5de03b9b13ce5369d5e1917d98d55f76c9fb7e02cfde79b5a34f7ff4888f7c37b4629028ad3632faccef79202867054278d7651ea824b16e4440156846c1b6c17e34248f4907f4f077a5
**Receiver’s data: SC’**	248	4361E9EE86FA4828E80EB2C39A418351417DA3E558A447C98E7C9A9E31D7A2D8CEEA034ED01C6EB35D8E9F9158B466808A7C902E6F7DB95DC2D6E332CB5F2BC4d2f3ce73717fc67c0ebd0938ddca2d76611b7726fcae1 cd6fcf8d3d97c5d7ed206eacbf5adbbe188dc319f9711d511d418fd38f4f8679d0358c854578c26ebf49daee3fb6129564dbbfe2af934e7cd6abe198dbfab097a168fbf36131bec99aba13c04f30c5ffae0a26bc22de38c5de03b9b13ce5369d5e1917d98d55f76c9fb7e02cfde79b5a34f7ff4888f7c37b4629028ad3632faccef79202867054278d7651ea824b16e4440156846c1b6c17e34248f4907f4f077a5
**RPRK**	32	620880abd1a3aac4981b18d72ebd129f735ff4e795e98b0702ed9a3e2098e7d7
**SC**_**3**_ **’**	32	79202867054278d7651ea824b16e4440156846c1b6c17e34248f4907f4f077a5
**Receiver’s decrypted data: SK’**	16	D65F878CE8E6DC3F2428382540845DDE
**Receiver’s decrypted data: Salt’**	16	e9e679e5b90a03fd918248f51f27d265
**Receiver’s decrypted data: M’**	60	48656c6c6f2c2074686973206973206d792062616e6b206163636f756e742e20506c65617365206b65657020697420636f6e666964656e7469616c21

### Benchmarking results of SM2, SM3, and SM4 cryptographic algorithms

Under identical experimental conditions, implemented exclusively using the Bouncy Castle cryptographic library and without utilizing CPU (Intel Core i9 9880H) hardware acceleration instructions, a comparative analysis of performance was conducted between the SM2, SM3, and SM4 cryptographic algorithms and their respective international counterparts: RSA-3072, SHA-256, and AES-256. To enhance the measurement accuracy of the experimental results, this study adopts byte (Byte) and nanosecond (ns) as the basic units of measurement. These high-precision time units and standardized data measurement units can more accurately reflect the system performance indicators and provide a reliable quantitative basis for subsequent data analysis.

### Performance testing results of SM2 and RSA-3072

Under the same conditions, in this study, for small data blocks not exceeding 32 bytes, a performance comparison test was conducted between the SM2 algorithm and the RSA-3072 algorithm. It consists of three parts: encryption and decryption, and key generation. The performance test results are shown in Table 3.

**Table 3:** The performance testing results of the SM2 and RSA-3072.

Data size (bytes)	SM2			RSA-3072		
	Encryption (ns)	Decryption (ns)	Key generation (ns)	Encryption (ns)	Decryption (ns)	Key generation (ns)
32	2154627	1591348	609266	97343	3500014	391766550
16	2142340	1580580	609266	96765	3492717	391766550
8	2163487	1587019	609266	96551	3493019	391766550

### Performance testing results of SM3 and SHA-256

This study compared the computational performance of the SM3 and SHA-256 algorithms in a standard experimental environment. The detailed results of the performance tests are presented in Table 4.

**Table 4:** The performance testing results of the SM3 and SHA-256.

Data size (bytes)	SM3	SHA-256
	Time (ns)	Time (ns)
8	394	445
16	403	456
32	414	469
64	773	850
128	1152	1261
256	1924	2097
512	3431	3729
1024	6451	6954
2048	12315	13462
4096	24289	26326
8192	48064	52279
16384	95225	103789
32768	189877	205891
65536	386191	411346
131072	759878	819850

### Performance testing results of SM4 and AES-256

Under the same experimental conditions, performance tests for encryption and decryption of the SM4 algorithm were conducted in comparison with the AES-256 algorithm. The experimental results are presented in Table 5.

**Table 5:** The performance testing results of SM4 and AES-256.

Data size (bytes)	SM4			AES-256		
	Encryption time (ns)	Decryption time (ns)	Total time (ns)	Encryption time (ns)	Decryption time (ns)	Total time (ns)
8	1721	1742	3464	2150	2287	4437
16	1941	1934	3874	2307	2424	4731
32	2160	2141	4300	2457	2537	4994
64	2597	2554	5151	2741	2770	5510
128	3348	3376	6724	3283	3262	6545
256	4950	4935	9885	4361	4192	8553
512	8170	8185	16354	6496	6058	12554
1024	14576	14490	29066	10731	9838	20569
2048	27509	27180	54689	19190	17193	36383
4096	53121	52718	105839	36226	32348	68574
8192	104157	103634	207790	69694	62042	131736
16384	206636	204806	411442	137587	121717	259303
32768	409587	407611	817198	272212	240724	512936
65536	817139	814137	1631276	539627	478569	1018196
131072	1630337	1624348	3254685	1072700	950883	2023583

### Evaluation results of security performance

The hybrid security framework proposed in this study, which integrates the SM2, SM3, and SM4 cryptographic algorithms with dynamic salt values, demonstrates comprehensive protection against mainstream security threats in instant messaging systems. Security performance evaluation results are shown in Table 6.

**Table 6:** Evaluation results of security performance.

Security mechanism	Information leakage	Credential theft	MITM attacks	Server attacks	Database attacks	Network sniffing
**Bidirectional authentication** [35]	●	●	●	●		●
**Decentralized architecture**				●	●	
**Hybrid message encryption**	●		●	●	●	●
**Ciphertext transmission and storage**	●	●	●	●	●	●

(● Indicates effective protection, blank indicates non-applicability).

## Discussion

Through the data analysis of the experimental results in Table 2, the experimental verification results, as shown in Table 7, were obtained.

**Table 7:** Confidentiality, integrity, and availability analysis results.

Sender’s data block name	Receiver’s data block name	Experimental verification results	Confidentiality, integrity, and availability results
M	M’	M = M’	Confidentiality and availability are ok
SK	SK’	SK = SK’	Confidentiality and availability are ok
SC3	SC3’	SC3 = SC3’	Integrity is ok
SC	SC’	SC = SC’	Confidentiality is ok

According to the experimental verification results in Table 7, the IMS secure communication scheme proposed in this paper achieves the design goals in all three core security dimensions. Firstly, by comparing and analyzing the consistency of the original plaintext data block (M, SK) at the sender’s end and the decrypted and restored data block (M’, SK’) at the receiver’s end (M ≡ M’ and SK ≡ SK’), it is confirmed that the system simultaneously meets the requirements of confidentiality and availability at the message transmission layer. No plaintext data leakage occurred during the communication process, and the data packets were complete and could be correctly parsed. Secondly, the verification value SC3 generated by the SM3 hash algorithm is completely consistent with the calculated value SC3’ at the receiver’s end (SC3 ≡ SC3’), which verifies that the integrity protection mechanism during data transmission can effectively resist MITM tampering attacks. Finally, through network packet capture analysis, it is confirmed that the encrypted data SC remains completely consistent with the received data SC’ at the receiver’s end during transmission (SC ≡ SC’). This result not only verifies the correctness of the encryption algorithm but also indicates that the system can ensure that sensitive information does not leak during transmission over public channels, meeting the confidentiality requirements of high-security-level instant messaging.

**Fig 9:**
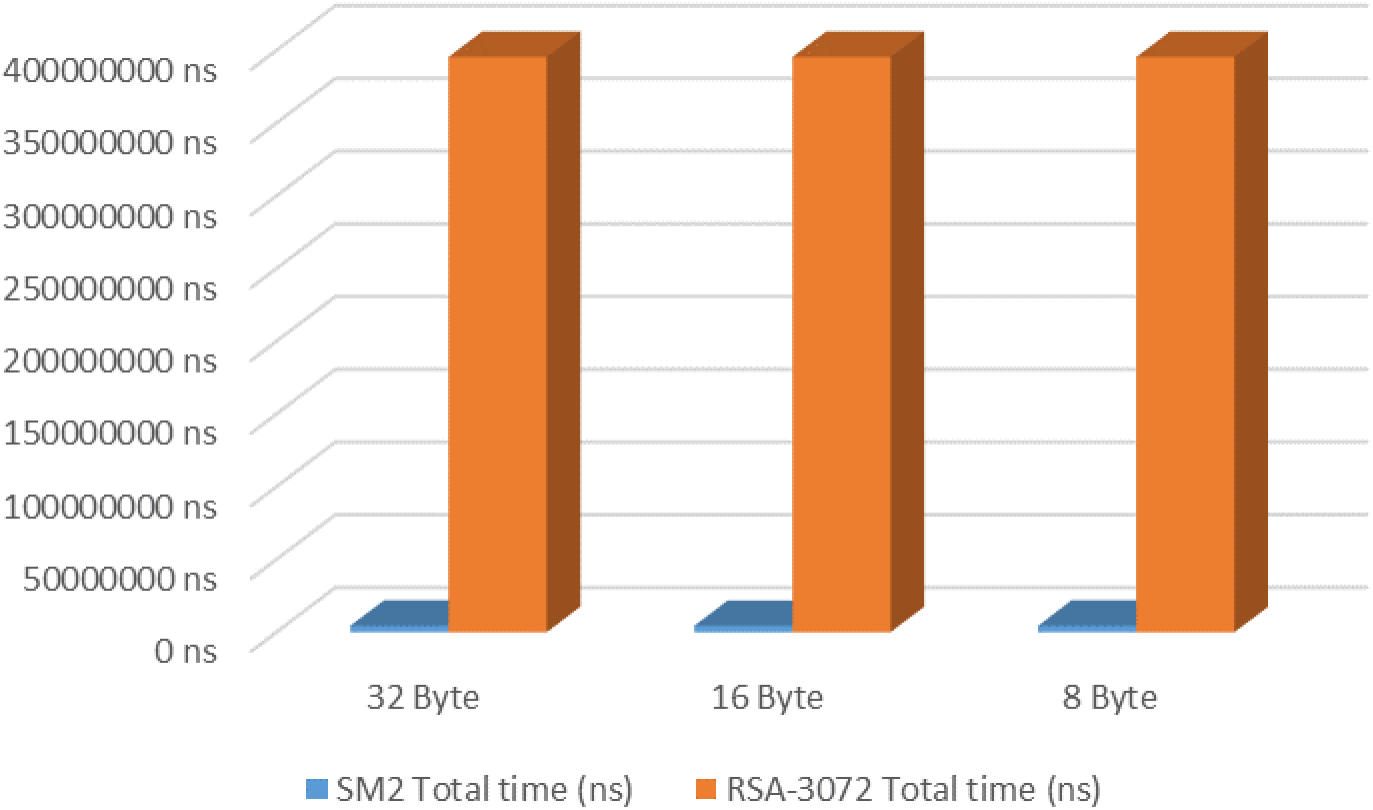
Overall performance comparison of SM2 and RSA-3072 algorithms.

Experimental analysis of the performance comparison between SM2 and RSA-3072 algorithms (Table 3) reveals significant operational disparities when processing data blocks ≤32 bytes. During the encryption and decryption test, each set of data undergoes 1,000 cycles of calculation. After the same 30 rounds of calculation, the obtained experimental data were sorted in descending order and processed using the median truncation method: the first 10 maximum values and the last 10 minimum values are removed, and the arithmetic mean of the 10 valid data points in the middle is calculated. Meanwhile, 1000 key pairs were calculated respectively for the SM2 and RSA-3072 algorithms. The median truncation method was also adopted for processing, and the arithmetic mean of the generation time of the middle 600 pairs of keys was taken. The cryptographic evaluation demonstrates that RSA-3072 exhibits superior encryption efficiency, with execution times ranging 96,551–97,343 ns, representing merely 4.55% (1/22) of SM2’s encryption duration (2,142,340–2,163,487 ns). Conversely, SM2 demonstrates a remarkable decryption performance advantage, operating at 1,580,580–1,591,348 ns compared to RSA-3072’s 3,492,717-3,500,014 ns, achieving approximately 2.2 × faster processing speed. The most pronounced performance divergence occurs during the key generation phase, where SM2 completes the operation in 609,266 ns versus RSA-3072’s 391,766,550 ns, exhibiting a 642:1 performance ratio. As illustrated in Fig 9, a comprehensive evaluation confirms SM2’s dominant performance characteristics in small-data cryptographic scenarios. For 32-byte data processing, SM2’s overall performance surpasses RSA-3072 by a factor of 90.78, primarily attributable to RSA’s computationally intensive key generation mechanism. In our proposed scheme, SM2 demonstrates particular efficacy in handling 16-byte session keys (SK) and 16-byte salt values, where rapid key deployment is essential. This performance advantage, coupled with equivalent security guarantees, establishes SM2 as an optimal cryptographic solution for lightweight IMS communication architectures.

**Fig 10:**
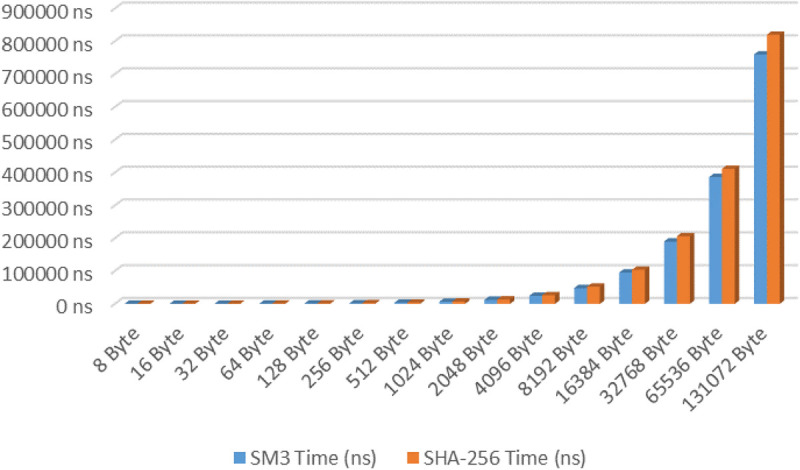
The performance comparison between SM3 and SHA-256.

Benchmark testing of hash algorithms in Table 4 demonstrates that SM3 exhibits significant efficiency advantages across varying data scales, as illustrated in Fig 10. Each algorithm was executed for 20,000 iterations, and 30 independent tests were conducted. To ensure robust data processing, a median-trimmed method was employed: after sorting the durations from the 30 test rounds, the top and bottom 10 extreme values were excluded, and the average of the remaining 10 rounds was calculated. When processing 8-byte data packets, SM3 achieves a processing time of 394 ns, representing an 11.5% throughput efficiency improvement over SHA-256 (445 ns). At the extended data volume of 128KB (131,072 bytes), SM3 maintains a 7.3% performance advantage with a processing duration of 759.9 µs. Experimental results confirm that compared to internationally prevalent algorithms, SM3 demonstrates superior operational suitability for IMS instant messaging scenarios, providing empirical support for its implementation in IMS systems.

**Fig 11:**
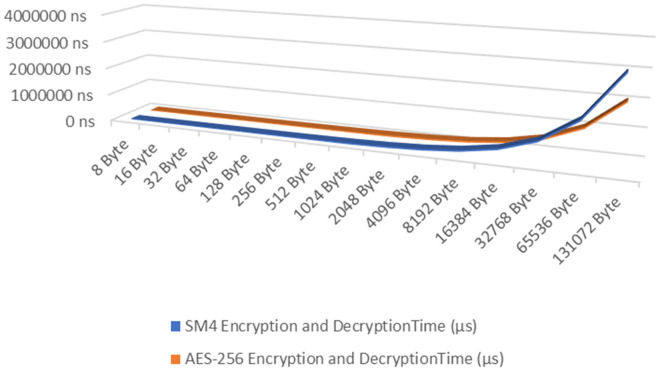
The performance comparison between SM4 and AES-256.

The experimental data presented in Table 5 illustrate the performance comparison between the SM4 and AES-256 encryption algorithms, as shown in Fig 11. Each dataset was processed in a loop for 10000 iterations. Following 30 rounds of computation, the results from these rounds were sorted, and the top 10 as well as the bottom 10 results were excluded. Only the average value of the middle 10 rounds of test outcomes was retained. The SM4 algorithm demonstrates significantly superior encryption and decryption performance compared to AES-256 within the data range of 8–64 bytes. For 8-byte data, the average time taken by SM4 is 1731.5 ns, which represents a reduction of 21.95% compared to AES-256’s time of 2218.5 ns; for 16-byte data, SM4 takes an average of 1937.5 ns, reducing the time by 18.10% relative to AES-256’s 2365.5 ns; at the 32-byte mark, SM4 requires an average of 2150.5 ns, showing a decrease of 13.88% when compared with AES-256’s time of 2497 ns; finally, for data sizes up to 64 bytes, SM4 averages at 2575.5 ns—6.53% less than AES-256’s duration of 2755.5 ns. Considering the overall performance from sizes ranging between 8 and 64 bytes, there exists a notable advantage in encryption and decryption times for the SM4 algorithm that spans from approximately 6% to 22% over its counterpart. While it is acknowledged that AES-256 exhibits higher throughput with larger datasets (greater than 512 bytes), SM4 proves more suitable for scenarios involving smaller amounts of data—particularly in fulfilling short message encryption requirements within IMS —making it ideally suited for real-time interactive applications.

According to Table 6 of the experimental evaluation results, the comprehensive protection effectiveness of the security framework based on the SM series hybrid algorithm in the instant messaging system has been verified. The proposed security scheme was evaluated and analyzed by using the Kali Linux network penetration testing method [36]. The multi-layer defense architecture effectively mitigates six categories of security threats through four interlocking security mechanisms. This framework achieves multi-dimensional security protection through the following technical features: Firstly, it adopts a two-way authentication mechanism based on national encryption standards. It has been measured that it can effectively resist MITM attacks and credential theft behaviors. Secondly, the innovative decentralized architecture design, through the deployment of distributed nodes, significantly reduces the risk of a single point of failure and enhances system availability. Thirdly, implement a full-link encryption strategy, covering the data transmission and storage links, to ensure the confidentiality and integrity of end-to-end communication; Finally, through the organic synergy of the SM2, SM3, and SM4 algorithms, a multi-level defense system was constructed. Each algorithm complemented the other’s strengths, resulting in a significant improvement in security while maintaining system performance. This hierarchical and progressive security architecture design provides a solution that takes into account both security and availability for instant messaging systems.

The obtained results have several practical implications for the deployment of secure instant messaging systems (IMS). First, the demonstrated efficiency improvements of SM2, SM3, and SM4 over traditional algorithms indicate that the proposed framework can be integrated into real-time communication platforms without introducing additional latency, thereby ensuring a seamless user experience. Second, the decentralized and certificateless design reduces reliance on centralized authentication servers, which not only mitigates single-point failure risks but also enhances system robustness against targeted attacks. Third, the client-side encryption in combination with the encrypt-before-store principle provides an additional layer of protection for sensitive data, which is particularly valuable in high-security domains such as finance, enterprise communication, and public services. Overall, these practical benefits underscore the potential of the proposed solution to strengthen the confidentiality, integrity, and availability of IMS in real-world applications.

Despite the demonstrated security and efficiency advantages of the proposed hybrid encryption framework in lightweight, real-time IMS applications, several limitations should be noted. The framework is primarily tailored for short-message scenarios, and its relatively high design complexity may lead to reduced performance when processing large data blocks or operating in resource-constrained environments. Future work will aim to optimize processing throughput for large data blocks and improve performance on resource-limited devices, while preserving the high security guarantees established in the current experiments.

## Conclusions

This study innovatively proposes a hybrid encryption security framework based on the SM2, SM3, and SM4 algorithms, significantly enhancing the security protection capabilities of IMS. Experimental results demonstrate that this framework successfully achieves triple guarantees of data confidentiality, integrity, and availability while maintaining communication efficiency. The proposed solution leverages the complementary advantages of the SM2, SM3, and SM4 algorithms. In scenarios involving small data sizes, it exhibits remarkable performance benefits. Specifically, the performance of the SM2 algorithm in key generation and decryption stages improves by 642 times and 2.2 times compared to RSA-3072; the processing speed of the SM3 hashing algorithm surpasses SHA-256 by 7.3% to 11.5%; furthermore, for encryption efficiency concerning small data blocks ranging from 8 to 64 bytes, the SM4 algorithm outperforms AES-256 by achieving an improvement of up to 22%. Through innovative decentralized architecture design and end-to-end full-link encryption strategies, this framework effectively mitigates security threats such as MITM attacks and data tampering while establishing an efficient end-to-end encrypted transmission system for IMS. Future research will refine the security scheme by optimizing processing throughput for large data blocks and enhancing performance in resource-constrained environments, ultimately strengthening cross-platform compatibility.
